# Use of artificial intelligence to assess genetic predisposition to develop critical COVID-19 disease: a comparative study of machine learning models

**DOI:** 10.1515/almed-2025-0073

**Published:** 2025-05-05

**Authors:** Salomón Martín Pérez, Flora Sanchez Jimenez, Sandra Fuentes Cantero, Marta Jímenez Barragan, Catalina Sanchez Mora, Juan M. Borreguero Leon, Arrobas Velilla Teresa, Agustín Valido Morales, Juan A. Delgado Torralbo, Antonio León Justel

**Affiliations:** 16582 Service of Clinical Biochemistry Virgen Macarena University Hospital Seville, Seville, Spain; Department of Clinical Laboratory Chemistry Rio Tinto General Hospital Huelva, Huelva, Spain; Unit of Pulmonology, Virgen Macarena University Hospital Seville, Seville, Spain

**Keywords:** machine learning, COVID-19, critical disease, artificial intelligence, genetic polymorphisms (SNPs), logistic regression

## Abstract

**Objectives:**

Early prediction of critical COVID-19 disease is crucial for an optimal clinical management. The objective of this study was to optimize predictive models for critical COVID-19 disease. Clinical data, laboratory data and genetic polymorphisms were integrated into AI models to compare the performance of different machine learning algorithms.

**Methods:**

Data from 155 inpatients were analyzed, 23 of whom developed critical disease. A univariate analysis was performed to assess potential correlations between seven SNPs, nine clinical variables and 10 laboratory parameters at admission.

**Results:**

Of the 7 SNPs, only three SNPs demonstrated a significant association with critical disase, namely: rs77534576, rs10774671 and rs10490770. The ensemble models exhibited the best performance: Random Forest (AUC=0.989), XGBoost (AUC=0.954) and AdaBoost (AUC=0.927). Variable importance varied across models, with age, C-reactive protein, heart diseases and the three SNPs being the most influential features. The predictive power of models improved with the integration of the three SNPs, as compared to previous studies where genetic data were not included. Internal validation confirmed the superiority and stability of the ensemble models.

**Conclusions:**

Machine learning models may help predict progression into critical COVID-19-disease. The predictive power of models improves when SNPs associated with COVID-19 severity are integrated with laboratory and clinical data. Prior to implementation in clinical practice, larger studies in different populations are needed to validate and support the generalization of these results.

## Introduction

The COVID-19 pandemic had a dramatic, long-lasting impact on global healthcare. This disease challenged the adaptability of health systems worldwide and disclosed deficiencies so far overlooked. There is limited evidence available on the underlying mechanisms governing the wide variability in COVID-19 severity across patients, ranging from mild/asymptomatic disease to critical disease [1]. The risk of mortality is determined by a combination of factors, including susceptibility to viral infection and predisposition to develop lung inflammation [2]. Interestingly, the severity of disease has changed significantly over time as a function of the prevalent viral strain, added to other factors, where immunization of the population has become crucial [3].

Artificial intelligence (AI) offers new opportunities and tools, having played a key role during the COVID-19 pandemic with applications in diagnosis, monitoring, contact tracing, drug and vaccine development, and in reducing the healthcare burden, thereby facilitating crisis surveillance and research efforts [4]. The global health crisis boosted cooperation among researchers worldwide, thereby enabling the rapid generation of crucial data on SARS-COV-2, including reference genomes [5] and the identification of genetic susceptibility factors. A range of international projects, including GWAS and whole exome studies [6], 7], were conducted to assess between-subject variability in susceptibility to the virus. These studies uncovered a potential association between different polymorphisms (SNPs) and genetic predisposition to develop critical disease [8], [9], [10].

To optimize the prediction of critical COVID-19 disease, the SNPs associated with severity were integrated with laboratory and clinical data in different machine learning models for a comparative study. Firstly, an analysis was performed to assess the association of candidate SNPs with disease severity. Then, only the SNPs found to be significantly associated with severity were incorporated into the models. The importance of disease-associated variables in each model was examined. Then, the contribution of the SNPs to each prediction was determined to examine the influence of these polymorphisms in prediction.

## Materials and methods

This study included patients older than 18 years admitted to the emergency department of Virgen Macarena University Hospital in Seville, Spain, with a diagnosis of COVID-19 confirmed by RT-PCR (reverse transcription polymerase chain reaction) in a Cepheid Xpert^®^ Xpress SARS-CoV-2 system between May 2020 and January 2021. Samples were collected by the hospital Biobank and sent to the laboratory for genome screening for SNPs. Informed consent was obtained from patients for use of their genetic material. This study was approved by the Institutional Review Board.

The study variable, critical COVID-19 disease, is defined as the occurrence of one or more of the following events during hospitalization: admission to the intensive care unit (ICU), need for invasive ventilation, or death. This definition, based on previous studies on severe COVID-19 outcomes, was used as a categorical variable (yes/no) for the primary outcome [11], 12]. This information was extracted from the electronic medical records of patients.

The predictive models were created on the basis of clinical data, laboratory data and SNPs. Clinical data were extracted from electronic medical records, including dichotomous variables (sex, radiological pulmonary findings, defined as images suggestive of pulmonary abnormalities on a radiography/CT scan; heart disease; hypertension; diabetes; autoimmune diseases, including lupus, rheumatoid arthritis, psoriasis and myasthenia gravis; tobacco use; and previous respiratory infections within the last month; and a continuous variable (age). Continuous laboratory variables, obtained from the first laboratory analysis at admission, included C-reactive protein, creatine kinase, D-dimer creatinine, lymphocyte count, alanine aminotransferase, platelet, urea, hemoglobin and lactate.

Prior to statistical analysis, an analysis of missing laboratory data at admission was performed. Missing values were imputed using the median of each variable.

The SNPs included rs10490770, located near the LZTFL1 and LOC107986083 genes on chromosome 3 [13], 14]; rs10774671, the OAS1 gene on chromosome 12; rs77534576, between the genes on chromosome 17 [13], 14]; rs2109069, in the DPP9 gene on chromosome 19 [15], 16]; rs74956615, near the FDX2 and RAVER1 genes on chromosome 9 [17]; and rs2834158, in the IFNAR2 gene on chromosome 21 [6], 18], all included in the PreMed-Covid19 study [19]. We added the SNP rs35705950, located in the MUC5B gene on chromosome 11 [20].

Samples were collected and frozen in the laboratory, and screening for polymorphisms was performed. Then, DNA was extracted from peripheral blood. Screening for genetic variants was performed by RT-PCR on a Cobas Z 480 (Roche Diagnostics GmbH) analyzer. The association between each polymorphism and critical disease was individually assessed using logistic regression. Four genetic inheritance models were considered: dominant, recessive, additive and codominant. Compliance with Hardy-Weinberg equilibrium was verified using the Chi-squared test prior to logistic regression analysis. Genotypes were coded according to each genetic inheritance model. The most adequate model for each SNP was determined by comparing the fitting of the co-dominant model with the other models, based on the likelihood ratio and the Akaike information criterion. Finally, a p<0.20 was established for a SNP to be included in the predictive models.

The totality of the variables available was included in all models. In machine learning models, automated variable selection was applied. In turn, the variables for the logistic regression model were selected based on statistical criteria.

### Logistic regression model

Firstly, quantitative variables were converted into binary variables (age, hemoglobin, platelets, lymphocytes, dimers, urea, creatinine, lactate dehydrogenase, alanine transaminase, creatine kinase, C-reactive protein), to facilitate clinical interpretation and implementation. Clear, optimal decision-making thresholds were established based on ROC curves and Youden statistics. Multicollinearity across predictive variables was assessed using the variance inflation factor (VIF). The variables with a VIF >5 were excluded. The individual association between each predictor and the outcome variable was assessed by univariate analysis. Univariate logistic regression models were adjusted for each variable. The variables with a prevalence of ≥5 % and those with a bilateral p<0.20 on univariate analysis were included in the multivariate logistic regression model.

### Machine learning models

For data pre-processing, continuous quantitative variables were standardized using the StandardScaler class from the scikit-learn library, which implements Z-score normalization During pre-processing, the dataset was split into 80 % for training and 20 % for testing, and SMOTE was applied to the training dataset to address class imbalance. To assess the robustness and stability of the models, two complementary internal validation approaches were used: 5-fold cross-validation and bootstrap validation with 1,000 iterations, using resampling with replacement.

The statistical analysis was carried out using Python, where pandas (v1.2.4) was employed for data handling; scikit-learn (v0.24.2) for developing predictive models and computing evaluation metrics; and imbalanced-learn (v0.8.0) to address class imbalance through SMOTE. The XGBoost model was implemented using the xgboost library (v1.4.2), while statistical inference was performed with statsmodels (v0.12.2). Finally, matplotlib (v3.4.2) was used to generate visual representations of the results.

Six models were evaluated, each based on a different approach. The K-Nearest Neighbors (KNN) algorithm classifies instances based on similarity to neighboring data points; Random Forest combines multiple decision trees to enhance accuracy and reduce overfitting; AdaBoost adjusts the weights of misclassified instances to improve model performance; XGBoost is known for its high efficiency and predictive power through gradient-boosted decision trees; Support Vector Machines (SVM) with a radial basis function (RBF) kernel aim to identify the optimal hyperplane for class separation; and Naive Bayes applies Bayes’ theorem under the assumption of feature independence. GridSearchCV was used in the training cohort to optimize hyperparameters.

### Model evaluation

The performance of each model was evaluated using multiple metrics, of which the area under the receiver operating characteristic curve (AUC) was the primary measure. The AUC assesses a model’s ability to discriminate between classes. Additional metrics included accuracy (proportion of correct predictions); precision (ratio of true positives to the total number of positive predictions); sensitivity (ratio of true positives to real positives); and F1 score (harmonic mean of precision and sensitivity). Feature importance to each model was analyzed using a permutation-based approach, which estimates the impact of each feature on model performance by randomly shuffling its values. Values were standardized to percentages to facilitate model comparison. Model coefficients were used for logistic regression to quantify the influence of each predictor in the outcome.

## Results

The study cohort included a total of 155 inpatients, of whom 23 progressed into critical disease. The variables analyzed were classified into two categories: quantitative and dichotomous. There were missing data for creatine kinase (CK) with 12 missing values (7.79 %); D-dimers and lactate dehydrogenase (LDH) with four missing values each (2.60 %); and C-reactive (PCR) with one missing value (0.65 %), which were imputed using the median value.

Dichotomous variables are summarized in Table 1. The alleles rs77534576, rs10490770 and rs10774671 were more frequent in critical patients, with frequencies being 17.4 %, 34.8 % and 21.7 % respectively, vs. 4.5 %, 18.9 % and 9.8 % in non-critical patients. Additionally, hypertension and heart diseases were more frequent in the critical group (36.4 % and 18.2 %, respectively), as compared to the non-critical group (36.4 % and 18.2 %, respectively). As many as 73.9 % of critical patients were admitted to the general ward, vs. 81.8 % of non-critical patients, most of whom were directly admitted to the intensive care unit. In contrast, differences were not as notable in other variables such as infection, autoimmune disease, diabetes, tobacco use, and radiological findings.

**Table 1:** Distribution of dichotomous variables.

Dichotomous variable	General (n=155)	Critical disease (n=23)	Non-critical disease (n=132)
rs77534576	10 (6.5 %)	4 (17.4 %)	6 (4.5 %)
rs10490770	33 (21.3 %)	8 (34.8 %)	25 (18.9 %)
rs10774671	18 (11.6 %)	5 (21.7 %)	13 (9.8 %)
Infection	4 (2.6 %)	1 (4.3 %)	3 (2.3 %)
Autoimmune disease	6 (3.9 %)	0 (0 %)	6 (4.5 %)
Hypertension	62 (40 %)	14 (60.9 %)	48 (36.4 %)
Diabetes	26 (16.8 %)	4 (17.4 %)	22 (16.7 %)
Heart disease	33 (21.3 %)	9 (39.1 %)	24 (18.2 %)
Tobacco use	19 (12.3 %)	3 (13 %)	16 (12.1 %)
Ward admission	125 (80.6 %)	17 (73.9 %)	108 (81.8 %)
Radiological findings	115 (74.2 %)	18 (78.3 %)	97 (73.5 %)

The Table shows the number and percentage of patients with specific characteristics in three groups: the general group (n=155); patients with critical disease (n=23); and patients without critical disease (n=132). Percentages were calculated from the total for each group.

Quantitative variables are displayed in Table 2. Median age was higher in critical patients (69 vs. 58 years). Hemoglobin values (14.6 g/dL) were similar in the two groups, with minimal values being lower in the critical group (12.7 vs.13.5 g/dL). Platelet count was slightly higher in the critical group (239 vs. 221 × 10ˆ3/µL). Dimer and creatinine concentrations were also higher in critical patients (585 vs. 486 ng/mL and 1 vs. 0.9 mg/dL, respectively). Lactate dehydrogenase values were slightly more elevated in critical patients (296 vs. 275 U/L), whereas creatine kinase was considerably higher in this group (111.5 vs. 75.5 U/L). Levels of C-reactive protein were significantly higher in critical patients (79 vs. 50.7 mg/L). Urea and lymphocyte concentrations were similar in the two groups. However, the latter were included for analysis, since our previous study [11] uncovered broader differences between groups.

**Table 2:** Distribution of quantitative variables.

Quantitative variable	General (n=155)	Critical disease (n=23)	Non-critical disease (n=132)
Age, years	59.5 [46–69]	69 [62–73.5]	58 [45–66]
Hemoglobin, g/dL	14.6 [13.4–15.4]	14.6 [12.7–15.4]	14.6 [13.5–15.4]
Platelets, × 10^3^/μL	222 [173–285.8]	239 [137.5–268]	221 [175.5–287]
Lymphocytes, × 10^3^/μL	1.3 [0.9–1.6]	1.3 [1–1.8]	1.2 [0.9–1.6]
Dimers, ng/mL	488 [337.2–835.2]	585 [390–977.5]	486 [335–776]
Urea, mg/dL	32 [25–41]	33 [28–45]	31 [25–41]
Creatinine, mg/dL	0.9 [0.8–1.1]	1 [0.8–1.2]	0.9 [0.7–1.1]
Lactate dehydrogenase, U/L	276 [209.2–342]	296 [200–361.5]	275 [211–338.5]
Alanine transaminase, U/L	31 [20–50]	27 [20.5–34.5]	32 [20–51.5]
Creatin kinase, U/L	79 [50–138]	111.5 [58.8–213]	75.5 [48–126.2]
C-reactive protein, mg/L	55.8 [25.5–102.2]	79 [57.2–133.9]	50.7 [22.2–96.9]

This Table displays the median and interquartile ranges [Q3-Q1] of different quantitative features in three groups of patients: the general group (n=155); patients with critical disease (n=155); and patients without critical disease (n=132).

### Analysis of polymorphisms

Only three SNPs were selected for inclusion in the predictive models. This selection was based on a thorough analysis based on HW equilibrium and comparison of inheritance models.

Six of the seven polymorphisms studied complied with HW equilibrium both in cases and controls (Supplementary Table 1). The SNPs selected included rs2834158, rs35705950, rs74956615, rs2109069, rs77534576 and rs10490770. Chi-squared values ranged from 0.01 to 2.46 *with* p*-*values >0.05, which excludes significant differences between the theoretical and true allelic frequencies. Of note, the polymorphism rs10774671 uncovered a significant deviation from HW equilibrium in the case group (χ^2^=7.99, p=0.005), whereas equilibrium was maintained in controls (χ^2^=1.86, p=0.173).

Comparison of each inheritance model with the co-dominant model (Supplementary Table 2) revealed that three SNPs met the p<0.20 criterion for inclusion in the predictive models. The three SNPs were rs77534576, for which an additive inheritance model was selected; rs10774671, which showed better fitting with a co-dominant model; and rs10490770 with an additive model,

### Models

**Figure 1:**
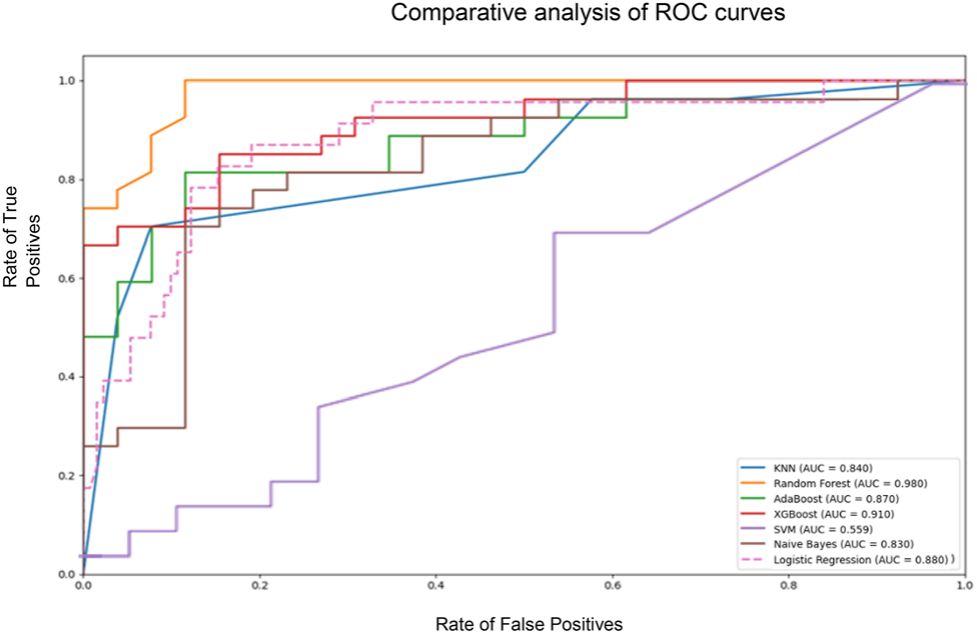
Comparative analysis of ROC curves for the different models. Comparative analysis of ROC curves for the different machine learning models. The graph shows the rate of true positives against the rate of false positives for each model. The area under the curve (AUC) is a measure of the model’s ability to distinguish classes, in this case, critical COVID-19 disease.

Machine learning models were superior to logistic regression models in predicting progression into critical COVID-19-disease. Figure 1 displays the ROC curves for each model, which demonstrate that ensemble learning models such as Random Forest (AUC=0.98), AdaBoost (AUC=0.87), and XGBoost (AUC=0.91), tended to have a superior performance in terms of AUC, as compared to individual models. Individual models included KNN (AUC=0.84); SVM (AUC=0.36); Naive Bayes (AUC=0.83); and Logistic Regression (AUC=0.88).

Table 3 displays all model evaluation metrics, of which area under the ROC curve (AUC) was the primary measure. Random Forest was the model with the best performance, as it showed the highest AUC (0.989), the highest accuracy (90.6 %), the second best precision (92.3 %), a high sensitivity (88.9 %), and the highest F1-Score (0.906), hence being significantly superior to traditional logistic regression in all metrics. XGBoost was the second model with the best AUC (0.954) and the highest precision (95.2 %). However, this model had a sensitivity as low as 74.1 %, below Random Forest. Its accuracy was 84.9 % and its F1-Score was 0.833, the second best values following Random Forest. AdaBoost had the third best AUC (0.927), with an accuracy of 81.1 %, a precision of 87.0 %, a sensitivity of 74.1 %, and a F1-Score of 0.800.

**Table 3:** Model evaluation metrics.

Model	Accuracy	Precision	Sensitivity	F1-score	AUC	VP	FP	FN	VN	Total
Random Forest	0.906	0.923	0.889	0.906	0.989	24	2	3	24	53
XGBoost	0.849	0.952	0.741	0.833	0.954	20	1	7	25	53
AdaBoost	0.811	0.870	0.741	0.800	0.927	20	3	7	23	53
Logistic regression	0.877	0.750	0.261	0.387	0.881	6	2	17	28	53
Naive Bayes	0.735	0.685	0.888	0.774	0.830	24	11	3	15	53
KNN	0.679	0.639	0.852	0.730	0.823	23	13	4	13	53
SVM	0.528	0.538	0.519	0.528	0.559	14	12	13	14	53

Metrics for several classification models evaluated in terms of accuracy, precisión, sensitivity, F1-Score, area under the curve (AUC) and confusion matrix values (TP, FP, FN, TN). The models evaluated include KNN, Random Forest, AdaBoost, XGBoost, SVM, Naive Bayes and Logistic Regression.

Following application of VIF, the following variables were included in the logistic regression model: age (≥65 years); genetic markers rs10774671, rs10490770 and rs77534576; presence of heart diseases; and arterial hypertension. The laboratory parameters included in the model were lymphocyte count (≥1.94 × 10ˆ3/µL); levels of creatine kinase (≥102 U/L); C-reactive protein (≥62.50 mg/L); D-dimers (≥942 ng/mL); creatinine (≥1.06 mg/dL); lactate dehydrogenase (≥296 U/L); and urea (≥27 mg/dL). Although the logistic regression model had the second best accuracy (87.7 %), it achieved an AUC as low as 0.881, below the three ensemble models. The logistic regression model also showed the poorest sensitivity (26.1 %) and the lowest F1-Score (0.387) of all models, although with moderate precision (75 %).

The remaining models exhibited variable results. Naive Bayes achieved an AUC of 0.830, having the second highest sensitivity (88.8 %), following SVM, but with a lower precision (68.5 %). KNN achieved an AUC of 0.823, with a high sensitivity (85.2 %) but with the second poorest precision (63.9 %). SVM, despite having the highest sensitivity (92.5 %), had the lowest AUC (0.559) and the poorest accuracy (50.9 %), with suggests a potential overfitting to the positive class.

Remarkable differences were observed in feature importance between machine learning models (Table 4) and the logistic regression model (Table 5, Supplementary Figure 1). Random Forest identified the rs10774671 (14.14 %) polymorphism, platelets (12.12 %), and rs77534576 (10.10 %) and rs10490770 (7.07 %) as the most important features. XGBoost identified heart diseases (37.25 %), creatine kinase (11.76 %) and C-reactive protein (10.46 %) as the most relevant predictors. AdaBoost prioritized C-reactive protein (14.59 %), D-dimers (11.35 %) and radiological findings (10.81 %). According to KNeighbors, the most important features were D-dimers (54.10 %), platelets (17.21 %) and alanine aminotransferase (9.84 %). SVM showed a strong preference for D-dimers (81.82 %), followed by lactate dehydrogenase (13.64 %). Naive Bayes identified age (9.14 %), hypertension and alanine aminotransferase (8.60 % each) as the factors with the highest influence. Finally, logistic regression identified age ≥65 years (13.59 %), the rs10774671 polymorphism (12.80 %) and a lymphocyte count ≥1.94 (10.65 %) as the most important factors.

**Table 4:** Percentage of feature importance by IA-based model.

Feature	Random Forest, %	KNeighbors, %	AdaBoost, %	XGBoost, %	SVM, %	Naive Bayes, %
Age	6.06	0.00	1.62	7.19	9.57	9.14
C-reactive protein	3.03	6.56	14.59	10.46	17.38	1.08
Heart diseases	7.07	0.00	1.62	37.25	0.35	5.38
Hypertension	1.01	0.00	2.70	9.15	12.77	8.60
Creatine kinase	5.05	1.64	2.16	11.76	4.26	2.15
rs10774671	14.14	0.00	2.16	3.92	12.41	5.91
Creatinine	8.08	0.00	7.03	0.00	0.00	4.84
D-dimers	0.00	54.10	11.35	5.23	0.00	4.84
Lymphocytes	2.02	0.00	1.08	0.00	0.71	6.45
Alanine aminotransferase	6.06	9.84	10.27	0.00	0.00	8.60
Platelets	12.12	17.21	5.41	0.00	2.48	0.00
Urea	0.00	0.82	9.19	0.00	3.55	2.69
Hemoglobin	0.00	0.00	0.00	2.61	6.03	1.08
rs10490770	7.07	0.00	5.41	3.27	1.77	1.61
Lactate dehydrogenase	2.02	9.84	1.08	0.65	4.26	2.15
Diabetes	10.10	0.00	0.54	0.00	0.00	5.38
rs77534576	10.10	0.00	3.24	1.31	10.99	6.99
Sex, male	1.01	0.00	5.41	2.61	1.77	5.91
Radiological	0.00	0.00	10.81	0.00	0.00	2.69
Ward admission	4.04	0.00	3.24	4.58	1.77	0.00
Tobacco use	1.01	0.00	1.08	0.00	0.00	0.54
Infection	0.00	0.00	0.00	0.00	13.12	8.06
Autoimmune	0.00	0.00	0.00	0.00	2.13	5.91

This Table shows the relative importance of each feature within the models Random Forest, K-Neighbors, AdaBoost, XGBoost, SVM, and Naive Bayes.

**Table 5:** Percentage of feature importance in the logistic regression model.

Feature	Coefficient	Importance, %	Odds ratio	p-Value
Age (≥65)	1.16	13.59	5.81	<0.001
Gen (rs77534576)	1.10	12.80	4.39	0.04
Lymphocytes (≥1.94)	0.91	10.65	2.54	0.10
Creatine kinase (≥102)	0.90	10.56	2.85	0.03
C-reactive protein (≥62.50)	0.84	9.77	3.92	0.01
Gen (rs10774671)	0.75	8.71	2.52	0.15
Gen (rs10490770)	0.66	7.69	2.05	0.14
Heart diseases	0.59	6.93	2.87	0.05
Hypertension	0.55	6.38	2.69	0.04
D dimers (≥942)	0.34	3.95	2.73	0.05
Creatinine (≥1.06)	0.31	3.60	2.42	0.08
Lactate dehydrogenase (≥296)	0.27	3.20	1.89	0.17
Urea (≥27)	0.19	2.18	2.32	0.19

This Table summarizes the relative importance of each feature within the logistic regression model. The cut-off point established for each quantitative feature is shown next to their name. The column Coefficient represents the magnitude and direction of the association between the feature and the outcome. The column Importance (%) refers to the relative contribution of each feature to the model. The Odds ratio shows the case-control likelihood ratio; and the p value indicates the statistical significance of the association. A cut-off point of p<0.20 was established for inclusion in the model.

SNPs showed a variable importance according to the model. The rs10774671 polymorphism was especially important for Random Forest (14.14 %) and logistic regression (12.80 %). The rs77534576 polymorphism was found to be the most relevant on the Random Forest model (10.10 %) and had a moderate importance in the logistic regression model (7.68 %). The rs10490770 polymorphism had a moderate importance in the Random Forest (7.07 %) and the logistic regression (8.71 %) model.

### Internal validation

Bootstrapping internal validation with 100 iterations revealed generalized improvements with respect to the original results (Supplementary Table 3). The Random Forest model retained its top-rank position, with an accuracy of 95.6 % ± 3.0 % and an AUC of 0.994 ± 0.008, closely followed by the XGBoost and AdaBoost models, which showed significant improvements with respect to the initial model (94.4 % ± 3.6 % y 93.2 % ± 3.8 % respectively). KNN also experienced a significant improvement (80.6 % ± 5.8 %), whereas the logistic regression model showed a similar performance, with high variability in precision and sensitivity. Naive Bayes showed stability. Finally, although SVM performance improved slightly, it was instable. In general, the ensemble models were superior and showed higher stability.

## Discussion

The most relevant finding in this study is that AI-based models exhibited a higher predictive power for COVID-19 progression into critical disease, as compared to the classic logistic regression model. Specifically, the ensemble models were superior, with the Random Forest model showing the best performance, with an AUC of 0.989, followed by XGBoost with 0.954 and AdaBoost with 0.927, as compared to the logistic regression model, with an AUC of 0.881. The models exhibited distinctive patterns in predicting critical COVID-19, with each algorithm emphasizing different predictive factors. Random Forest revealed that elevated levels of platelets and the presence of the rs10774671, rs77534576 and rs10490770 polymorphisms increase the risk for progression into critical disease, with an importance of 12.12 %, 14.14 %, 10.10 % and 7.07 %, respectively. XGBoost detected the presence of heart diseases as the factor with the strongest predictive value (37.25 %), followed by elevated levels of creatine kinase (11.76 %) and C-reactive protein (10.46 %). AdaBoost pointed to elevated levels of C-reactive protein (14.59 %) and D-dimers (11.35 %) as the factors indicating a higher risk. Consistency among multiple models in identifying these factors, especially SNPs and inflammatory markers, supports their validity as robust predictors of critical COVID-19, although their relative weight varies depending on the algorithm used.

The evaluation of genetic predisposition revealed that only three of the seven SNPs were associated with critical COVID-19 disease in our cohort of patients (rs77534576, rs10774671 and rs10490770). All models, except for SVM and KNN, identified these SNPs as significant factors, although with varying relative importance across models.

The ensemble models Random Forest, AdaBoost and XGBoost demonstrated an optimal performance. This finding supports their suitability for predictive purposes, as they identify subtle but crucial patterns that identify high-risk patients. Interestingly, the KNN model, despite its simplicity, exhibited a respectable performance. Logistic regression achieved a high overall accuracy, but a low sensitivity. However, its easy interpretability still represents a significant advantage in the clinical setting.

The integration of SNPs associated with disease severity enhances prediction of COVID-19 progression into critical disease. A previous study performed by our research group [11] on critical COVID-19 disease where SNPs were not included, the logistic regression model achieved an AUC of 0.850. In contrast, the AUC for the current logistic regression model, which included SNPs, increased to 0.881. These improvements suggest that the inclusion of genetic data has enhanced the predictive power of the model.

Analysis of the Hardy-Weinberg equilibrium revealed that, except for rs10774671 in the case group, all SNPs were in equilibrium both in cases and controls. This result supports the validity of our finding that genetics are involved in the risk for progression into critical disease. Of the seven SNPs analyzed, three were statistically associated with critical COVID-19: rs77534576, rs10774671 and rs10490770. For rs77534576 and rs10490770, an additive inheritance model was identified as the one with the best performance, thus suggesting a cumulative effect of each allele on the risk for developing severe disease. This is consistent with the results obtained by Yi Lin et al. [21], where rs77534576 was one of the SNPs associated with the risk for hospitalization and the development of very severe COVID-19-related respiratory symptoms. In relation to rs10774671, our analysis favoured a co-dominant model, thus indicating different effects for each genotype. This result is aligned with the previous studies conducted by El Yousfi et al. [13] and Huffman et al. [14], which highlighted the protective effects of the G allele of rs10774671 against severe COVID-19 disease. A deviation from Hardy-Weinberg equilibrium was observed for rs10774671. A lower relative proportion of subjects in the case group had the protective allele, as compared to controls. In relation to rs10490770, our findings highlight its major role in predicting COVID-19 severity, consistently with the study by Nakanishi et al. [22]. Conversely, our results contradict the non-significant results reported by Prajjval P et al. [23] for the Indian population. These inconsistencies emphasize the relevance of considering genetic diversity in the population for the interpretation of results. The rs35705950 SNP was not found to be significant, which is in disagreement with Van Moorsel et al., who demonstrated that the T allele of MUC5B rs35705950 confers protection against severe COVID-19 [20].

Comparative analysis of the importance of each variable unveiled that age, C-reactive protein, heart diseases and the three SNPs (rs10490770, rs10774671 and rs77534576) were relevant in all models, except for SVM and KNN, although with varying importance. Inconsistencies in feature importance across models emphasize the complexity of the problem. This finding suggests that a multiple-model approach would provide a more robust understanding of predictive factors. The identification of these genetic markers not only improves our knowledge of the underlying mechanisms of susceptibility to severe COVID-19, but also paves the way to the use of personalized medicine in the management of the pandemic.

The integration of these genetic factors with clinical and laboratory variables in predictive models is a significant step forward towards a more precise, personalized risk stratification. However, genetic predisposition is only a piece of the puzzle, and its interpretation should be considered from a broader perspective including environmental factors, comorbidities and individual immune response. Differences in variable importance across models demonstrate the need for using multiple approaches to gain a more comprehensive understanding of the problem.

This study has some limitations, including a relatively small sample size of 155 patients, which may lead to overfitting in complex models. This occurs due to the imbalance between the number of observations and the number of predictors, thus increasing the likelihood that the model captures sample-specific noise rather than true population-level relationships. Additionally, it was not specified whether baseline laboratory values were obtained at ED admission or at ICU admission, which may influence the interpretation of results due to variations in biomarkers over time. Although cross-validation was applied to mitigate these problems, internal and external validation of these findings in a larger diverse cohort is essential prior to their implementation in clinical practice. The limited evidence available in the literature on specific methods for establishing associations between SNPs and particular variables or establishing the type of dominance model is a methodological challenge common to genetic association studies.

This study demonstrates the superiority of machine learning algorithms, especially ensemble models, in predicting critical COVID-19 disease. The use of SNPs in combination with laboratory and clinical variables enhances the predictive power of models. Of the seven SNPs analyzed, a statistically significant association was observed between three SNPs and critical COVID-19 disease, namely: rs77534576, rs10774671 and rs10490770. As a result, these SNPs were integrated in the predictive algorithms, thereby suggesting a genetic predisposition to develop critical COVID-19 disease. Further studies based on larger, more diverse populations are needed to validate and generalize the results obtained in this study, including external validation in independent populations.

## Supplementary Material

Supplementary Material

Supplementary Material

Supplementary Material

Supplementary Material

## References

[1] Halacli B, Yildirim M, Kaya EK, Ulusoydan E, Ersoy EO, Topeli A (2024). Chronic critical illness in critically ill COVID-19 patients. Chronic Illn.

[2] Wu F, Zhao S, Yu B, Chen YM, Wang W, Song ZG (2020). A new coronavirus associated with human respiratory disease in China. Nature.

[3] Martinón-Torres F (2022). Vacunación pediátrica frente al COVID-19 y a pesar del COVID-19. Pediatr (Barc).

[4] Vaishya R, Javaid M, Khan IH, Haleem A (2020). Artificial Intelligence (AI) applications for COVID-19 pandemic. Diabetes Metab Syndr.

[5] Ganna A (2020). The COVID-19 host genetics initiative, a global initiative to elucidate the role of host genetic factors in susceptibility and severity of the SARS-CoV-2 virus pandemic. Eur J Hum Genet.

[6] Pairo-Castineira E, Clohisey S, Klaric L, Bretherick AD, Rawlik K, Pasko D (2021). Genetic mechanisms of critical illness in COVID-19. Nature.

[7] Ellinghaus D, Degenhardt F, Bujanda L, Buti M, Albillos A, Invernizzi P (2020). Genomewide association study of severe covid-19 with respiratory failure. N Engl J Med.

[8] Karjalainen J, Liao RG, Neale BM, Daly M, Ganna A, COVID-19 Host Genetics Initiative (2021). Mapping the human genetic architecture of COVID-19. Nature.

[9] Karjalainen J, Stevens C, Neale BM, Daly M, Ganna A, COVID-19 Host Genetics Initiative (2022). A first update on mapping the human genetic architecture of COVID-19. Nature.

[10] Ferreira LC, Gomes CEM, Rodrigues-Neto JF, Jeronimo SMB (2022). Genome-wide association studies of COVID-19: connecting the dots. Infect Genet Evol.

[11] Martin S, Fuentes S, Sanchez C, Jimenez M, Navarro C, Perez H (2021). Development and validation of a laboratory-based risk score to predict the occurrence of critical illness in hospitalized patients with COVID-19. Scand J Clin Lab Invest.

[12] Guan WJ, Ni ZY, Hu Y, Liang WH, Ou CQ, He JX (2020). Clinical characteristics of coronavirus disease 2019 in China. N Engl J Med.

[13] El Yousfi FZ, Haroun AE, Nebhani C, Belayachi J, Askander O, El Fahime E (2024). Prevalence of the protective OAS1 rs10774671-G allele against severe COVID-19 in Moroccans: implications for a North African Neanderthal connection. Arch Virol.

[14] Huffman JE, Butler-Laporte G, Khan A, Pairo-Castineira E, Drivas TG, Peloso GM (2022). Multi-ancestry fine mapping implicates OAS1 splicing in risk of severe COVID-19. Nat Genet.

[15] Velavan TP, Pallerla SR, Rüter J, Augustin Y, Kremsner PG, Krishna S (2021). Host genetic factors determining COVID-19 susceptibility and severity. EBioMedicine.

[16] Safari M, Tavakoli R, Aghasadeghi M, Tabatabaee Bafroee AS, Fateh A, Rahimi P (2024). Study on the correlation between DPP9 rs2109069 and IFNAR2 rs2236757 polymorphisms with COVID-19 mortality. Nucleosides Nucleotides Nucleic Acids.

[17] Fink-Baldauf IM, Stuart WD, Brewington JJ, Guo M, Maeda Y (2022). CRISPRi links COVID-19 GWAS loci to LZTFL1 and RAVER1. EBioMedicine.

[18] Fricke-Galindo I, Martínez-Morales A, Chávez-Galán L, Ocaña-Guzmán R, Buendía-Roldán I, Pérez-Rubio G (2022). IFNAR2 relevance in the clinical outcome of individuals with severe COVID-19. Front Immunol.

[19] Dopazo J, Maya-Miles D, García F, Lorusso N, Calleja MÁ, Pareja MJ (2021). Implementing personalized medicine in covid-19 in andalusia: an opportunity to transform the healthcare system. J Pers Med.

[20] van Moorsel CHM, van der Vis JJ, Duckworth A, Scotton CJ, Benschop C, Ellinghaus D (2021). The MUC5B promoter polymorphism associates with severe COVID-19 in the European population. Front Med.

[21] Lin Y, Jiang B, Cai Y, Luo W, Zheng C, Zhu X (2023). The causal relationship between COVID-19 and increased intraocular pressure: a bidirectional two-sample Mendelian randomization study. Front Public Health.

[22] Nakanishi T, Pigazzini S, Degenhardt F, Cordioli M, Butler-Laporte G, Maya-Miles D (2021). Age-dependent impact of the major common genetic risk factor for COVID-19 on severity and mortality. J Clin Investig.

[23] Singh PP, Srivastava A, Sultana GNN, Khanam N, Pathak A, Suravajhala P (2021). The major genetic risk factor for severe COVID-19 does not show any association among South Asian populations. Sci Rep.

