# Supplementary material for: Use of artificial intelligence to assess genetic predisposition to develop critical COVID-19 disease: a comparative study of machine learning models
Source: Adv Lab Med. 2025 May 5;6(2):181–9. doi: 10.1515/almed-2025-0073 (PMC12107411; doi:10.1515/almed-2025-0073)
Supplement: Supplementary file 1 — Supplementary Material [file j_almed-2025-0073_suppl_001.docx]

**Supplementary Figure 1.** Percentage of feature imporntace for the Logistic Regression model.


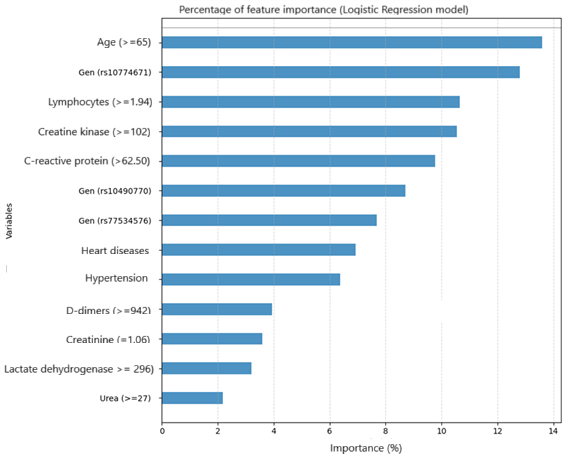


This graph shows the relative importance of each feature within the logistic regression model. The cut-off point established for each quantitative variable is also shown.
