# Supplementary material for: Use of artificial intelligence to assess genetic predisposition to develop critical COVID-19 disease: a comparative study of machine learning models
Source: Adv Lab Med. 2025 May 5;6(2):181–9. doi: 10.1515/almed-2025-0073 (PMC12107411; doi:10.1515/almed-2025-0073)
Supplement: Supplementary file 2 — Supplementary Material [file j_almed-2025-0073_suppl_002.docx]

**Supplementary Table 1**. HW equilibrium metrics for cases and controls.

|  | **CASES** | | | **CONTROLS** | | |
| --- | --- | --- | --- | --- | --- | --- |
| **Polymorphism** | **CHI2** | **P-VALUE** | **HW equilibrium** | **CHI2** | **P-VALUE** | **HW equilibrium** |
| **rs2834158** | 1.308 | 0.253 | SI | 0.469 | 0.494 | YES |
| **rs35705950** | 2.457 | 0.117 | SI | 0.011 | 0.917 | YES |
| **rs74956615** | 0.048 | 0.827 | SI | 0.357 | 0.550 | YES |
| **rs2109069** | 1.431 | 0.232 | SI | 0.083 | 0.773 | YES |
| **rs77534576** | 0.198 | 0.656 | SI | 0.072 | 0.789 | YES |
| **rs10774671** | 7.987 | 0.005 | NO | 1.855 | 0.173 | YES |
| **rs10490770** | 0.195 | 0.659 | SI | 0.668 | 0.414 | YES |

This Table shows *p* values and Chi-squared values for each SNP considered and specifies whether each SNP is in HW equilibrium for cases and controls.
