# Supplementary material for: Use of artificial intelligence to assess genetic predisposition to develop critical COVID-19 disease: a comparative study of machine learning models
Source: Adv Lab Med. 2025 May 5;6(2):181–9. doi: 10.1515/almed-2025-0073 (PMC12107411; doi:10.1515/almed-2025-0073)
Supplement: Supplementary file 3 — Supplementary Material [file j_almed-2025-0073_suppl_003.docx]

**Supplementary Table 2**. Analysis of dominance for the SNPs considered

| **SNP** | **model** | ***P* value** | **AIC** | **LRT_P_Value** | **Best fitting** | *P* **< 0.20** |
| --- | --- | --- | --- | --- | --- | --- |
| **rs2834158** | **Codominant** | 0.407 | 134.048 | - | Codominant | No |
|  | **Dominant** | 0.473 | 133.333 | 0.257 |  |  |
|  | **Recessive** | 0.390 | 133.108 | 0.303 |  |  |
|  | **Additive** | 0.851 | 133.812 | 0.184 |  |  |
| **rs35705950** | **Codominant** | 0.464 | 134.313 | - | Recessive | No |
|  | **Dominant** | 0.985 | 133.847 | 0.216 |  |  |
|  | **Recessive** | 0.240 | 132.467 | 0.695 |  |  |
|  | **Additive** | 0.721 | 133.724 | 0.235 |  |  |
| **rs74956615** | **Codominant** | 0.853 | 133.812 | - | Codominant | No |
|  | **Dominant** | 0.853 | 133.812 | 1.000 |  |  |
|  | **Recessive** | - | 131.847 | 0.853 |  |  |
|  | **Additive** | 0.855 | 133.812 | 1.000 |  |  |
| **rs2109069** | **Codominant** | 0.620 | 134.889 | - | Codominant | No |
|  | **Dominant** | 0.586 | 133.551 | 0.416 |  |  |
|  | **Recessive** | 0.577 | 133.535 | 0.422 |  |  |
|  | **Additive** | 0.880 | 133.824 | 0.334 |  |  |
| **rs77534576** | **Codominant** | 0.051 | 133.805 | - | Additive | Yes |
|  | **Dominant** | 0.051 | 133.805 | 1.000 |  |  |
|  | **Recessive** | - | 135.614 | 0.051 |  |  |
|  | **Additive** | 0.038 | 133.805 | 1.000 |  |  |
| **rs10774671** | **Codominant** | 0.117 | 131.561 | - | Codominant | Yes |
|  | **Dominant** | 0.597 | 133.568 | 0.045 |  |  |
|  | **Recessive** | 0.132 | 131.577 | 0.156 |  |  |
|  | **Additive** | 0.706 | 133.707 | 0.042 |  |  |
| **rs10490770** | **Codominant** | 0.358 | 133.790 | - | Additive | Yes |
|  | **Dominant** | 0.170 | 131.963 | 0.677 |  |  |
|  | **Recessive** | 0.421 | 133.200 | 0.235 |  |  |
|  | **Additive** | 0.136 | 131.794 | 0.950 |  |  |
