# Supplementary material for: Use of artificial intelligence to assess genetic predisposition to develop critical COVID-19 disease: a comparative study of machine learning models
Source: Adv Lab Med. 2025 May 5;6(2):181–9. doi: 10.1515/almed-2025-0073 (PMC12107411; doi:10.1515/almed-2025-0073)
Supplement: Supplementary file 4 — Supplementary Material [file j_almed-2025-0073_suppl_004.docx]

**Supplementary table 3.** Internal validation metrics.

| **Model** | **Accuracy** | **Precision** | **Sensitivity** | **F1-Score** | **AUC** |
| --- | --- | --- | --- | --- | --- |
| **KNN** | 0.806 ± 0.058 | 0.756 ± 0.082 | 0.905 ± 0.068 | 0.820 ± 0.059 | 0.904 ± 0.041 |
| **Random Forest** | 0.956 ± 0.030 | 0.956 ± 0.042 | 0.958 ± 0.045 | 0.956 ± 0.031 | 0.994 ± 0.008 |
| **AdaBoost** | 0.932 ± 0.038 | 0.926 ± 0.057 | 0.939 ± 0.052 | 0.931 ± 0.040 | 0.964 ± 0.032 |
| **XGBoost** | 0.944 ± 0.036 | 0.940 ± 0.053 | 0.948 ± 0.052 | 0.943 ± 0.038 | 0.986 ± 0.016 |
| **SVM** | 0.562 ± 0.095 | 0.585 ± 0.206 | 0.568 ± 0.321 | 0.514 ± 0.203 | 0.619 ± 0.183 |
| **Naive Bayes** | 0.726 ± 0.068 | 0.693 ± 0.095 | 0.825 ± 0.116 | 0.745 ± 0.073 | 0.824 ± 0.062 |
| **Logistic Regression** | 0.868 ± 0.060 | 0.535 ± 0.435 | 0.248 ± 0.238 | 0.312 ± 0.266 | 0.837 ± 0.11 |

Internal validation metrics for several classification models. Means and standard deviations (±) are shown for five metrics: accuracy, precision, sensitivity, F1-Score and area under the curve (AUC). The models evaluated include KNN, Random Forest, AdaBoost, XGBoost, SVM, Naive Bayes and Logistic Regression.
